# A single amino acid in the viral capsid makes a major contribution to the interaction between human norovirus GII.6 and the bacterial Psl exopolysaccharide

**DOI:** 10.1128/aem.02282-25

**Published:** 2026-06-22

**Authors:** Xiang Li, Haoyuan Tian, Yongjie Wang, Yongxin Yu

**Affiliations:** 1 College of Food Science and Technology, Shanghai Ocean University 608357, Shanghai, China; 2 Laboratory for Marine Biology and Biotechnology, Qingdao Marine Science and Technology Center, Qingdao, China; 3 Laboratory of Quality and Safety Risk Assessment for Aquatic Products on Storage and Preservation (Shanghai), Ministry of Agriculture and Rural Affairs, Shanghai, China; Michigan State University, East Lansing, Michigan, USA

**Keywords:** human noroviruses, Psl exopolysaccharide, P2 subdomain, V297 site, binding

## Abstract

Human noroviruses are a leading cause of foodborne gastroenteritis worldwide, and oysters are a major vector for their transmission. Previous work has shown that the Psl exopolysaccharide (EPS) from oyster-derived *Pseudomonas composti* strain ODT-54 binds to human norovirus GII.6, but the molecular mechanism remains unknown. Here, we combined molecular docking, dynamics simulations, Molecular Mechanics Poisson-Boltzmann Surface Area calculations, and site-directed mutagenesis to investigate this interaction. Our results suggest that the composition and conformation of the B loop contribute importantly to EPS-mediated bioaccumulation, and that residue valine 297 (V297) in the B loop of the GII.6 (KX752057.1) P domain is an important determinant of Psl binding. Our findings provide residue-level evidence that the composition and conformation of the B loop, particularly residue V297, contributes to the interaction between human norovirus GII.6 and the bacterial Psl exopolysaccharide.

**IMPORTANCE:**

The persistence of human noroviruses in environmental and food matrices can be facilitated by their interaction with bacterial surface components, such as exopolysaccharides (EPS). However, the molecular basis of norovirus-bacteria interactions remains poorly understood. In this study, we demonstrate that the GII.6 norovirus capsid protein specifically binds to the Pseudomonas-derived Psl EPS via a key residue, valine 297, located in the hypervariable B loop of the P2 subdomain. This interaction is genotype-specific, with GII.6 exhibiting significantly stronger binding than the epidemic GII.4 strain. Our findings reveal a previously unrecognized mechanism of viral environmental persistence mediated by bacterial EPS and highlight the role of capsid loop flexibility and residue-level variation in shaping norovirus-host-environment interactions. This work provides a structural and dynamic framework for understanding norovirus ecology and may inform future strategies for mitigating viral contamination in food and water systems.

## INTRODUCTION

Human norovirus is a positive-sense, single-stranded RNA virus with a genome of approximately 7.5–7.7 kilobases, containing three major open reading frames (ORFs). ORF1 encodes a nonstructural polyprotein that is cleaved into mature proteins responsible for viral replication. ORF2 and ORF3 are translated from a subgenomic RNA, encoding the major structural protein VP1 and the minor capsid protein VP2, respectively (1, 2). The capsid is assembled from 90 VP1 dimers, with each monomer consisting of a conserved shell (S) domain and a variable protruding (P) domain (3). The P domain is further divided into P1 and P2 subdomains. The P2 subdomain, which forms the outermost surface of the virion, is the most hypervariable region and contains binding sites for host ligands, including histo-blood group antigens (HBGAs), bile acids, and sialic acid (4–6).

Noroviruses are highly diverse and are classified into at least 10 genogroups and 49 genotypes. Among them, GII.4 has been the predominant genotype driving global epidemics (7, 8). In contrast, GII.6 strains are typically associated with sporadic infections (9). Notably, GII.6 infections occur independently of secretor status, suggesting they utilize host attachment factors distinct from HBGAs (10).

Human noroviruses can bind to extracellular polymeric substances on specific bacterial surfaces *in vitro* (11–13). We previously isolated *Pseudomonas composti* (ODT-54) from oyster digestive tissue and found that its surface exopolysaccharides (EPS), Psl, binds to the GII.6 genotype but shows only weak affinity for GII.4 (14). Psl is a repeating pentasaccharide composed of three D-mannose, one D-glucose, and one L-rhamnose residue, linked via 1,3-glycosidic bonds (15). This marked difference in binding affinity prompted us to investigate its structural basis.

In this study, we elucidate the molecular mechanism underlying the specific recognition of the bacterial Psl polysaccharide by the GII.6 norovirus P domain. Using an integrated approach of molecular docking, molecular dynamics (MD) simulations, Molecular Mechanics Poisson-Boltzmann Surface Area (MM-PBSA) binding free energy calculations, and site-directed mutagenesis, we systematically evaluated the contributions of key amino acid residues in Psl binding. Our results suggest that valine 297 (V297) is an important determinant of binding affinity. These findings provide atomic-level insight into the interaction between norovirus GII.6 and bacterial EPS and suggest that such interactions may contribute to viral environmental persistence and transmission.

## MATERIALS AND METHODS

### Expression and purification of norovirus P protein

The encoding sequence of the GII.6 P protein (residues 224–540, GenBank accession number KX752057.1; numbering according to this reference) was synthesized by GenScript Biotech (Nanjing, China) and inserted into the pET-28a(+) expression vector between the *Nde*I (upstream) and *Xho*I (downstream) restriction sites, with an N-terminal His tag (16). The GII.4 P protein used in this study was prepared previously. Briefly, the coding sequence for the GII.4 P protein Den Haag 2006b variant (residues 225–531, GenBank accession number LC177653.1) was cloned into the pET-30a expression vector (14). Before expression, the sequence was optimized for codon usage in *Escherichia coli*, and then the expression plasmid was introduced into *E. coli* BL21(DE3) cells via a heat-shock transformation method (17). Site-directed mutants (A294S and V297A) were constructed by commercial gene synthesis based on the same plasmid backbone and then subjected to the same cloning and expression procedures.

The transformed strain was inoculated into LB liquid medium and cultured until the OD₆₀₀ reached 0.5–0.6. Protein expression was then induced with 1 mM isopropyl β-D-1-thiogalactopyranoside at 16°C for 22 hours. Bacterial cells were harvested by centrifugation at 12,000 rpm for 10 minutes, and the solubility of the expressed protein was assessed by SDS-PAGE. Subsequently, western blot analysis was performed using a polyclonal antibody against the GII.6 P protein to confirm the expression of the N-terminal His-tagged fusion protein (14). The target protein was then purified using an ÄKTA Pure 150 system equipped with a 1 mL HisTrap Excel affinity chromatography column. The purity of GII.6, GII.6 A294S, and GII.6 V297A proteins was analyzed using ImageJ software (https://imagej.nih.gov/ij), with calculated purities of 89%, 95%, and 92%, respectively. The BCA assay further showed that the final concentrations of the GII.6 P and GII.6 V297A proteins were 3 mg/mL, whereas those of GII.6 A294S were 2.5 mg/mL.

### Extraction and purification of bacterial Psl exopolysaccharide

The Psl exopolysaccharide-overexpressing strain ODT-54 *psl*(+) was constructed from previous work (18). The mutant strain was inoculated into 20 mL of LB broth supplemented with 10 g/L L-arabinose and cultured at 37°C with shaking at 180 rpm for 18 hours until the OD₆₀₀ reached 0.6–0.8. The culture supernatant was then collected by centrifugation at 10,000 rpm for 30 minutes at 4°C.

To precipitate polysaccharides, proteins, and nucleic acids, precooled absolute ethanol was added to the supernatant to a final concentration of 80% (vol/vol), followed by incubation at 4°C for 24 hours (19). The precipitate was then collected by centrifugation at 15,000 rpm for 30 minutes at 4°C and resuspended in PBS buffer containing 10 mM MgCl₂ and 1 mM CaCl₂. To eliminate residual nucleic acids, DNase I (10 U/mL) and RNase A (100 µg/mL) were added to the solution and incubated at 37°C for 15 minutes. Protein impurities were then digested by adding proteinase K (100 µg/mL) and incubating at 55°C for 2 hours. Finally, the enzyme-treated mixture was heated at 80°C for 30 minutes to inactivate and denature the enzymes.

The sample was subjected to dialysis (molecular weight cutoff: 3.5 kDa) to remove inorganic salts and other small molecules, followed by lyophilization. The crude extracellular extract was further purified by DEAE-FF anion exchange chromatography (Xiamen Sanyi Technology, Xiamen, China) and size-exclusion chromatography using a Tiderose GF75 column (Suzhou Tiderose Biotechnology, Suzhou, China). Target polysaccharides were separated based on molecular size, shape, and surface charge (20), and the purified fractions were lyophilized to obtain Psl polysaccharide powder. The lyophilized products were screened with anti-Psl antibodies to identify Psl-enriched fractions. To further assess the homogeneity of the purified sample, high-performance gel permeation chromatography (HPGPC) was additionally performed. The resulting Psl-enriched fractions were then used for downstream experiments.

### Evaluation of the binding between bacterial Psl and norovirus P protein by ELISA

The binding affinities of four P proteins—GII.6 wild-type, GII.6 A294S mutant, GII.6 V297A mutant, and GII.4—toward purified extracellular polysaccharide Psl isolated from bacterial surfaces were evaluated using a modified ELISA protocol as described in our previous study (14).

Flat-bottom 96-well microplates were coated with 100 μL of norovirus P protein solution (5 μg/mL) and incubated overnight at 4°C. After incubation, the plates were washed three times with PBS-T buffer (PBS containing 0.05% Tween-20) to remove unbound protein. Subsequently, 200 μL of blocking buffer (10% BSA in PBS-T) was added to each well and incubated at 37°C for 3 hours to block nonspecific binding sites. After blocking, 100 μL of Psl-containing extracellular material solution (5 μg/mL) was added to each well and incubated at 37°C for 2 hours to allow binding. Following incubation with the carbohydrate ligands, 100 μL of anti-Psl IgG1 monoclonal antibody (1:500 dilutions; Creative Biolabs, UK) was added and incubated at 37°C for 2 hours. Unbound primary antibody was removed by washing the plate three times with PBS-T. Next, 100 μL of HRP-conjugated goat anti-human IgG secondary antibody (1:2,000 dilution; Abcam, USA) was added and incubated at 37°C for 1–2 hours. Wells were then washed five times with PBS-T to eliminate residual secondary antibody. Signal development was performed using an EL-TMB substrate kit (Sangon Biotech, Shanghai, China), and absorbance was measured at 450 nm using a microplate reader. All experimental conditions were tested in triplicate to ensure reliability and reproducibility.

### Homology modeling

The three-dimensional structures of the P domains from norovirus GII.6 (GenBank accession number KX752057.1), GII.4 (LC177653.1), and other GII.6 variants were constructed using homology modeling via the SWISS-MODEL online server (https://swissmodel.expasy.org/). Specifically, the GII.6 structure was modeled using the crystallographic template 7YQB, while the GII.4 structure was based on 4X07. All generated models achieved global model quality estimation scores above 0.8, indicating high structural quality and reliability (21). Site-directed mutagenesis of GII.6 was performed using the mutagenesis tool in PyMOL version 2.5.2 (https://www.pymol.org/) by substituting specific amino acid residues to generate the corresponding mutant structures. All models were subsequently visualized, refined, and analyzed using PyMOL version 2.5.2.

### Structure prediction of the Psl monomeric unit

The molecular structure of the Psl monomeric unit was modeled according to the reported monosaccharide composition of *Pseudomonas aeruginosa* PAO1 (22). The initial two-dimensional structure was drawn using ChemDraw (https://www.perkinelmer.com/category/chemdraw) and subsequently converted into a three-dimensional model in Chem3D (https://www.perkinelmer.com/category/3D). Energy minimization was performed in Chem3D using its built-in MM2 force field. In all subsequent molecular docking and molecular dynamics simulations, the Psl was represented by this modeled monomeric unit.

### Molecular docking

Molecular docking was performed using AutoDock Vina 1.5.6 (https://github.com/ccsb-scripps/AutoDock-Vina) to predict the binding conformations of the norovirus P protein with the Psl. The ligand was preprocessed by adding all hydrogen atoms and defining rotatable bonds, while the protein receptor was also hydrogenated and treated as a rigid macromolecule. Both the ligand and receptor were saved in PDBQT format using AutoDockTools.

Crystallographic studies have shown that the P2 subdomain is located on the outermost surface of the viral capsid and represents a key region mediating interactions with host receptors (23). For molecular docking, the intact P dimer was used as the receptor structure, and the docking grid box was centered on one of its P2 subdomains to encompass the carbohydrate-binding site. Specifically, the grid center was set at coordinates (28, –6, –4) for the GII.6-Psl docking and (–14, –22, –3) for the GII.4-Psl docking. The search space was defined as a cubic grid box with dimensions of 30 × 30 × 30 Å³, allowing the ligand to explore conformational space within 10 Å of the grid center. The grid spacing was adjusted to ensure sufficient coverage of the binding interface and to include residues surrounding the predicted binding pocket.

For each ligand, nine binding poses were generated in a single docking run, and the entire docking procedure was independently repeated three times to ensure reproducibility. The pose with the lowest predicted binding energy and a physically reasonable orientation was selected for subsequent molecular dynamics simulations and binding free energy calculations. All docked complexes were carefully examined in PyMOL (24) to confirm correct orientation within the binding site and to eliminate sterically unfavorable poses.

### Molecular dynamics simulations and binding free energy calculations

This study optimized and refined the molecular dynamics simulation protocol based on our previous work (25). The ligand structure output in PDBQT format from molecular docking was imported into Avogadro 1.2.0 (https://avogadro.cc/) for hydrogen addition and subsequently saved as a mol2 file (26). Topology files for the ligand were generated using Sobtop v.1.0 (http://sobereva.com/soft/Sobtop), based on the General Amber Force Field parameters (27). The protein topology was generated using the pdb2gmx tool in GROMACS 2023 (https://www.gromacs.org/), with the Amber ff14SB force field applied to the norovirus P domain (28).

The resulting protein-Psl complex was placed in a cubic simulation box with a minimum distance of 0.8 nm from any edge. The system was solvated using the TIP3P water model, followed by the addition of 0.15 mol/L NaCl to reproduce physiological ionic strength. Counterions were added to neutralize the total system charge (29).

Energy minimization was first performed to remove unfavorable contacts. This was followed by two-stage equilibration: an NVT phase using the V-rescale thermostat to gradually heat the system to 300 K under positional restraints on protein atoms, and an NPT phase using C-rescale isotropic pressure coupling at 1 bar, during which the restraints were progressively released to allow pressure adaptation.

A 100 ns production molecular dynamics simulation was then carried out in the NPT ensemble without any restraints. Prior to analysis, trajectory files were processed to remove periodic boundary conditions, and eliminate global translational and rotational motions. Root-mean-square deviation (RMSD) and root-mean-square fluctuation (RMSF) analyses were performed to assess the overall structural stability and the flexibility of key loop regions.

To further investigate the binding mechanism between the norovirus P domain and the Psl polysaccharide, binding free energy calculations were performed using the gmx_MMPBSA tool (https://valdes-tresanco-ms.github.io/gmx_MMPBSA/dev/) (30). The final 10 ns of the equilibrated trajectory—corresponding to the RMSD-stable region—was extracted for energy averaging. Per-residue free energy decomposition was conducted to identify key contributors to the interaction, and hydrogen bond analysis was performed to characterize the binding interface.

### Amino acid diversity analysis at position 297 in GII.6 noroviruses

A total of 1,427 norovirus nucleotide sequences were retrieved from the GenBank nucleotide database using the keyword “Norovirus GII.6” (as of 22 October 2025). Among them, 311 sequences containing the complete VP1 coding region were downloaded in FASTA format. To remove redundancy, CD-HIT v.4.8.1 (http://weizhong-lab.ucsd.edu/cdhit/) was used with a sequence identity threshold set at 99% (31), resulting in 86 non-redundant full-length VP1 sequences. Multiple sequence alignment of the 86 sequences was performed using Geneious software (https://www.geneious.com), and the aligned file was subsequently analyzed using WebLogo3 (http://weblogo.threeplusone.com/) to visualize sequence conservation and variation patterns, thereby revealing the evolutionary characteristics of the VP1 domain (32).

## RESULTS

### Structural comparison between GII.6 and GII.4 P proteins

To compare the structural characteristics of norovirus genotypes GII.6 and GII.4, structure-based sequence alignment and 3D superposition were performed. Overall, the two genotypes shared 62% sequence identity, with the S domain being highly conserved (78%) and the P domain less conserved (51%).

**Fig 1:**
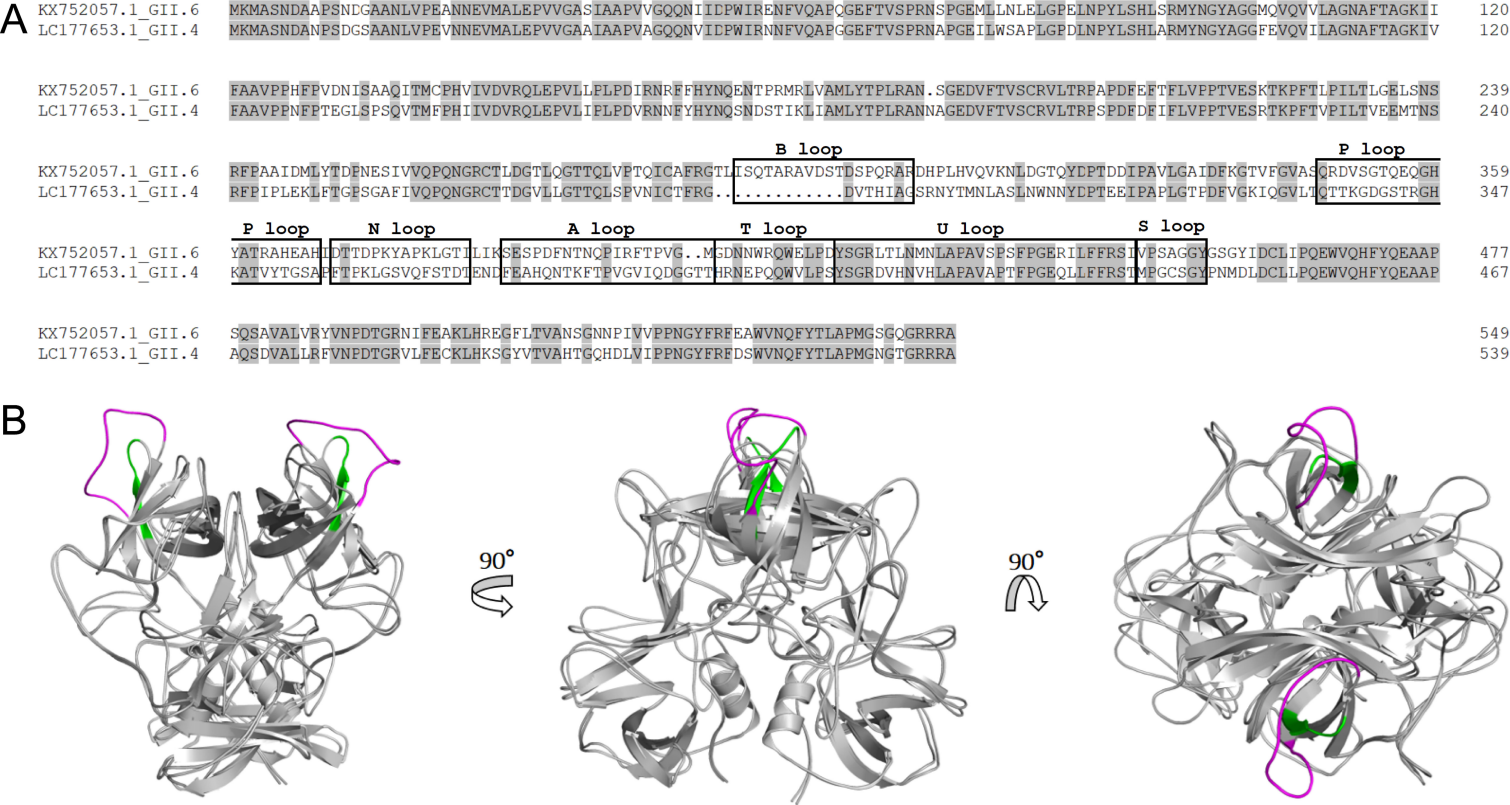
Structural comparison of GII.6 and GII.4 P proteins. (**A**) Amino acid sequence alignment of VP1 from GII.6 and GII.4; white regions indicate non-conserved residues, and major loop regions (B, S, P, T, N, A, and U loops) are labeled. (**B**) Structural superposition of GII.6 and GII.4 P proteins. The B loop of GII.6 (magenta) and B loop of GII.4 (green) are highlighted to illustrate their conformational differences.

Within the P domain, the B loop exhibited the most pronounced divergence, exhibiting only 16% sequence identity, with an additional 11 residue insertion unique to GII.6 (Fig. 1A). Structural superposition further revealed that the B loop of GII.6 adopts a flexible, protruding conformation distinct from the relatively compact B loop of GII.4 (Fig. 1B). These results highlight the B loop as the major structural distinction between these two genotypes.

### Docking analysis of GII.6 and GII.4 norovirus P proteins with Psl

Molecular docking analyses were performed using the P domain structures of the two genotypes in complex with the Psl ligand. The results showed that GII.6 exhibited a binding free energy of −7.0 kcal/mol with Psl, which was more favorable than that of the GII.4 wild-type (−6.6 kcal/mol).

**Fig 2:**
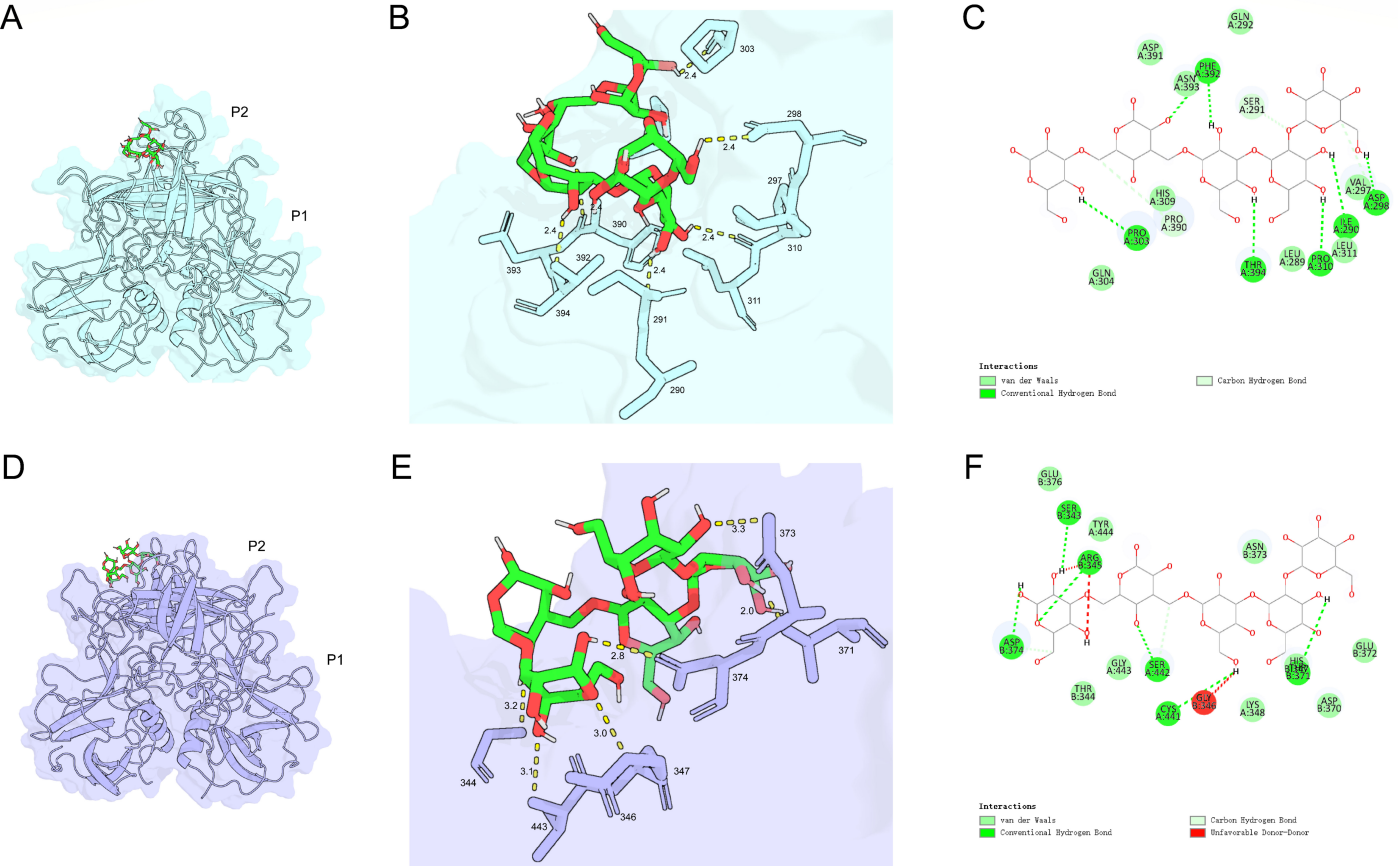
Structural basis of Psl recognition by GII.6 and GII.4 norovirus P domains. (**A**) Overall structure of the GII.6 P domain dimer in complex with the Psl polysaccharide. (**B**) Close-up view of the GII.6 binding pocket, highlighting key residues, including those in the B-loop region, that form directional hydrogen bonds with the hydroxyl groups of Psl. (**C**) Two-dimensional interaction network of the GII.6 P domain-Psl complex, illustrating hydrogen bonds, van der Waals contacts, and hydrophobic interactions at the binding interface. (**D**) Overall structure of the GII.4 P domain dimer bound to Psl, shown in cartoon representation with the polysaccharide depicted as sticks. (**E**) Enlarged view of the GII.4 binding interface, showing the spatial arrangement of residues involved in Psl recognition. (**F**) Two-dimensional interaction map of the GII.4 P domain-Psl complex, depicting hydrogen-bonding networks, van der Waals interactions, and hydrophobic contacts stabilizing the complex.

Structural visualization revealed that the P2 subdomain of GII.6 forms a complex and extensive hydrogen-bonding network with the repeating unit of the Psl polysaccharide at the binding interface (Fig. 2A). Among them, residues such as Ile290, Asp298, Pro303, Pro310, and Phe392 form multiple highly directional hydrogen bonds with the hydroxyl groups of Psl, thereby facilitating precise ligand recognition and stable anchoring (Fig. 2B). In addition, residues including Leu289, Val297, Leu311, Pro390, and Asn393 further stabilize the binding interface through hydrophobic interactions and van der Waals forces, providing the structural basis for the specific recognition and high-affinity binding of Psl by the GII.6 P domain (Fig. 2C).

The binding interface of GII.4 was more open and structurally relaxed compared with that of GII.6, forming a relatively sparse hydrogen-bonding network with the Psl polysaccharide (Fig. 2D). Only a few polar residues, such as Thr344, Gly346, Thr371, and Gly443, were involved in hydrogen bond formation. Most of these residues lack side chains capable of forming stable interactions, which may weaken molecular recognition and consequently reduce overall binding affinity (Fig. 2E). In addition, an unfavorable hydrogen-bond geometry between Gln431 and Psl was observed, which could introduce local steric repulsion and further destabilize the complex (Fig. 2F).

### Molecular dynamics insights into Psl recognition by norovirus P protein

To investigate the differences in conformational stability, structural flexibility, polysaccharide-binding ability, and key residue energy contributions between the GII.6 and GII.4 norovirus P domains, 100 ns MD simulations were performed for the two genotypes, followed by MM-PBSA binding free energy calculations and per-residue energy decomposition analysis.

**Fig 3:**
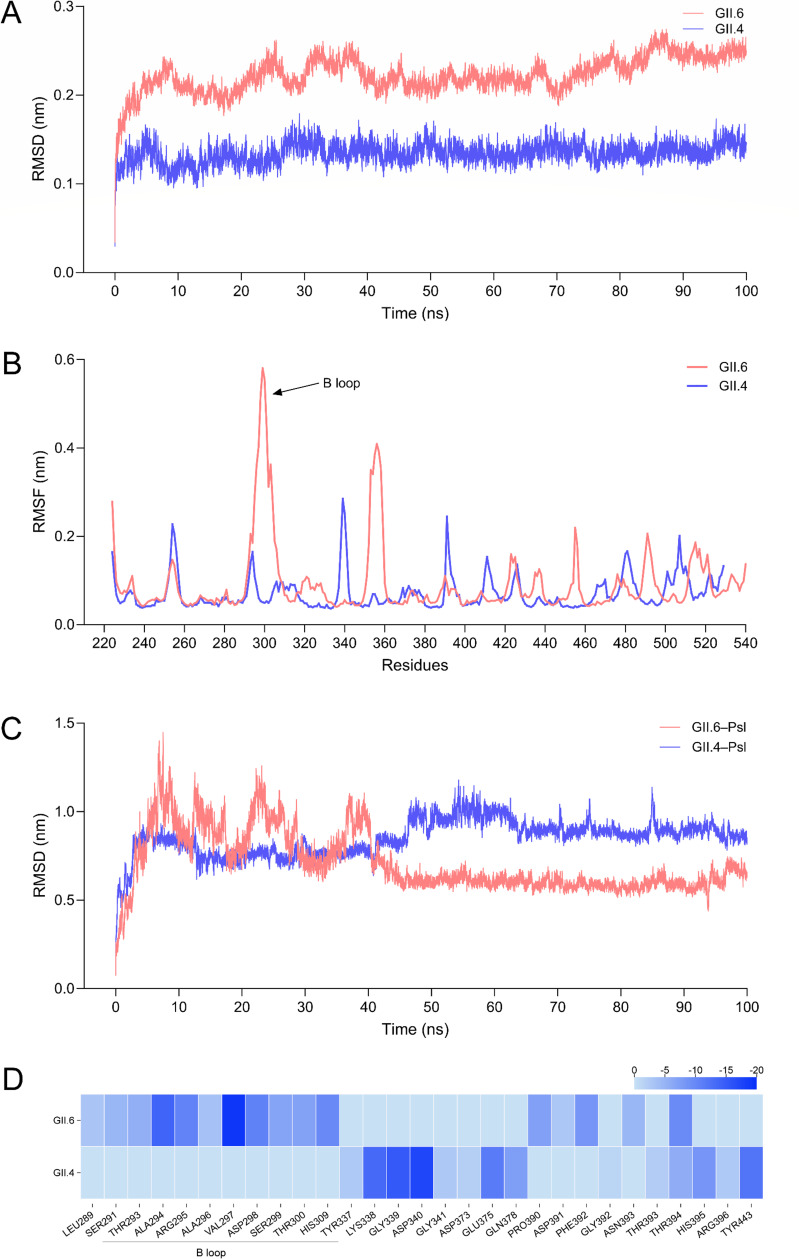
Molecular dynamics simulation analysis of GII.6 and GII.4 P proteins and their complexes with Psl. (**A**) RMSD trajectories of GII.6 and GII.4 wild-type P proteins over 100 ns molecular dynamics (MD) simulations. (**B**) Per-residue RMSF profiles of GII.6 and GII.4 P proteins, indicating local flexibility differences. Higher fluctuations in GII.6 were observed in the B-loop (residues 290–307) and P-loop (residues 348–365) regions. (**C**) RMSD trajectories of the GII.6-Psl and GII.4-Psl complexes. (**D**) Per-residue binding free energy decomposition analysis of GII.6 and GII.4 P proteins.

The RMSD of the GII.6 P domain stabilized around 0.21 nm during the simulation, whereas GII.4 remained consistently lower, around 0.12 nm, with smaller fluctuations (Fig. 3A). These results suggest that the GII.6 P domain possesses higher conformational flexibility, while GII.4 displays greater overall rigidity.

RMSF analysis further revealed residue-level differences in flexibility (Fig. 3B). GII.6 showed higher local fluctuations in several regions, particularly in residues 290–307 (B loop) and 348–365 (P loop), with peak RMSF values reaching approximately 0.6 nm and 0.4 nm, respectively. In contrast, GII.4 exhibited significantly lower flexibility in the corresponding regions, with peak RMSF values of only 0.18 nm and 0.23 nm. Notably, sequence and structural comparisons between the B- and P-loop regions of the two genotypes revealed substantial differences (Fig. 1). These observations suggest that the B loop may be a key structural determinant for enhanced binding, as its increased flexibility in GII.6 could facilitate conformational adaptation upon ligand engagement. Conversely, the absence or reduced flexibility of these regions in GII.4 may underlie its weaker binding performance.

To evaluate the stability of the P domain-Psl polysaccharide complexes, 100 ns MD simulations were also conducted for the complexes. The RMSD of the GII.6-Psl complex stabilized around 0.2 nm after approximately 40 ns, indicating a relatively stable conformation (Fig. 3C). In contrast, the GII.4-Psl complex exhibited a higher and more fluctuating RMSD of approximately 0.3 nm, suggesting a less stable and more dynamic binding interface. Binding free energy was calculated using the MM-PBSA method based on the final 10 ns (90–100 ns) of the equilibrated trajectories further supported these findings. The GII.6-Psl complex showed a more favorable binding free energy (−59.114 kcal/mol) compared to the GII.4-Psl complex (−50.853 kcal/mol), indicating stronger binding affinity of GII.6 to Psl.

Per-residue energy decomposition analysis identified key residues contributing to binding (Fig. 3D). Several residues in the B loop in GII.6, including Ala294, Arg295, Val297, and Asp298, contributed significantly (ΔG_binding < −10 kcal/mol), with Val297 showing the greatest energy contribution. In contrast, GII.4 lacked these key residues or exhibited weaker contributions at the corresponding positions, resulting in reduced binding affinity.

Overall, GII.6 exhibited superior structural flexibility, higher binding stability, and more favorable energetic contributions from key interface residues, providing a molecular explanation for its stronger interaction with the Psl polysaccharide compared to GII.4.

### Residues within the B-loop region involving Psl binding

**Fig 4:**
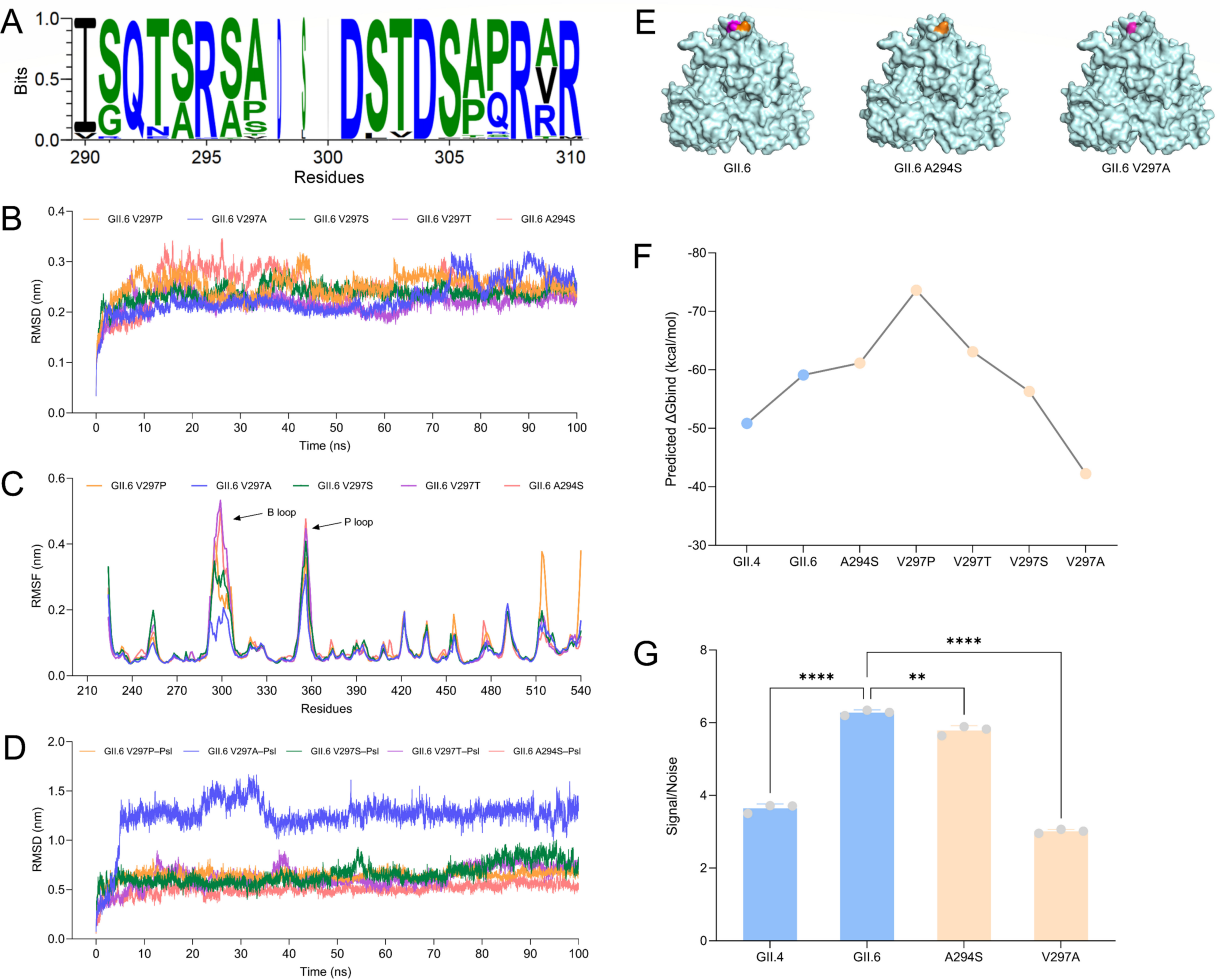
Evolution-based selection of representative B-loop mutants and validation of their effects on P domain structure and Psl binding. (**A**) Sequence logo of the B loop (residues 290–310) in GII.6 VP1 showing amino acid variability at position 297. (**B and C**) Conformational stability and flexibility of GII.6 wild-type and mutant P proteins evaluated by RMSD and RMSF analyses. (**D**) RMSD trajectories of GII.6 P-Psl complexes illustrating the structural stability of mutants upon ligand binding. (**E**) Surface representations of the wild-type, A294S, and V297A P proteins highlight the spatial positions of residues A294 and V297 within the ligand-binding interface. (**F**) Binding free energies (ΔG_bind) of GII.4, GII.6 wild-type, and GII.6 single mutants (A294S, V297P, V297T, V297S, and V297A) calculated using the MM-PBSA method. (**G**) Experimental ELISA results showing relative signal-to-noise (S/N) ratios for GII.4, GII.6, A294S, and V297A P proteins; data represent mean ± SD, *n* = 3 (**P < 0.01, ****P < 0.0001).

To further elucidate the roles of key amino acid residues within the B-loop region in mediating Psl binding, we first performed a WebLogo-based sequence conservation analysis of the B loop in GII.6 norovirus P proteins (Fig. 4A). The results revealed that Arg295 and Asp298 are highly conserved in this region. At position 297, amino acids including Val, Ala, Ser, Pro, and Trp were observed, whereas at position 294, Ala and Ser were present. Based on these sequence characteristics, we designed and constructed four single-point mutants at position V297 (V297A, V297S, V297P, and V297T) and one single-point mutant at position A294 (A294S). Each mutant was subjected to 100 ns MD simulations to systematically evaluate their effects on protein conformational stability, local flexibility, and binding affinity with Psl.

The RMSD analysis of the protein structures (Fig. 4B) indicated that the A294S, V297S, and V297T mutants exhibited conformational stability most closely resembling that of the wild-type GII.6, with RMSD values consistently maintained between 0.20 and 0.23 nm. V297P mutation showed slightly higher but still relatively stable RMSD trajectories, suggesting good structural stability. In contrast, the V297A mutant displayed noticeable conformational fluctuations, with RMSD values approaching 0.30 nm, implying possible local structural disturbances.

RMSF analysis further clarified the effects of these mutations on local protein flexibility (Fig. 4C). In the B-loop region (residues 290–307), all mutants exhibited varying degrees of flexibility changes. Notably, the V297A mutation significantly decreased flexibility in this region, while A294S, V297P, and V297S showed only slight rigidity reduction or localized disturbances, which may influence the conformational fit induced during Psl binding. In the wild-type GII.6, the β-branched Val297 forms a protruding hydrophobic bulge that helps maintain a convex and tightly packed apex of the B loop (Fig. 4E). Substitution with alanine reduces the side-chain volume, effectively flattening the apex, increasing local solvent exposure, and diminishing the hydrophobic contact area. In contrast, the A294S mutation induces only a subtle reshaping of the B-loop contour, leading to minor adjustments in local packing without disrupting the overall convex architecture.

Further RMSD analysis of the protein-Psl complexes revealed a pronounced decline in binding stability for the V297A mutant upon interaction with the Psl repeating unit (Fig. 4D). Its ligand RMSD (fit on the protein) rapidly increased and stabilized at 1.3 nm–1.5 nm, indicating that the Psl molecule exhibited significant displacement from the initial binding interface. In contrast, the A294S, V297S, V297P, and V297T mutants maintained RMSD profiles comparable to the wild type, suggesting stable ligand binding and complex formation.

To assess the binding affinity changes caused by these mutations, binding free energies were calculated based on the last 10 ns (90-100 ns) of the MD trajectories (Fig. 4F). The V297P mutant exhibited the lowest binding free energy (−73.621 kcal/mol), indicating the strongest binding affinity among all variants. Compared with the wild type (−59.114 kcal/mol), the V297T mutant (−63.089 kcal/mol) showed an enhanced binding affinity. The A294S mutant (−61.156 kcal/mol) fell within the same significance range as the wild type, suggesting no significant difference in binding. The V297S mutant (−56.333 kcal/mol) exhibited a slight decrease in affinity, whereas the V297A mutant showed a markedly higher binding free energy (−42.254 kcal/mol), indicating a substantial loss of binding affinity toward Psl.

### *In vitro* binding of Psl with wild-type and mutant GII.6 P proteins

To verify the effect of key B-loop residue mutations on Psl-binding affinity, we expressed and purified the wild-type GII.6 P protein and its mutants (A294S and V297A). The Psl exopolysaccharide used in the binding assays was purified as described above and further characterized by HPGPC. The ratio of weight-average molecular weight (Mw) to number-average molecular weight (Mn) was close to 1, indicating a relatively concentrated molecular weight distribution and good sample homogeneity (Fig. S1).

Subsequently, ELISA assays were performed to evaluate the binding abilities of these proteins to *P. composti-*derived Psl polysaccharides (Fig. 4G). The results showed that the wild-type GII.6 P protein exhibited the strongest binding capacity, followed by the A294S mutant. In contrast, the V297A mutant displayed a markedly reduced binding signal, indicating that this mutation significantly impaired Psl recognition. Moreover, the GII.4 P protein exhibited weaker binding to Psl than the wild-type GII.6 protein but significantly stronger binding than the V297A mutant, further highlighting the influence of genotype-specific structural differences on ligand recognition.

### Binding affinity analysis of GII.6 norovirus variants with Psl

**Fig 5:**
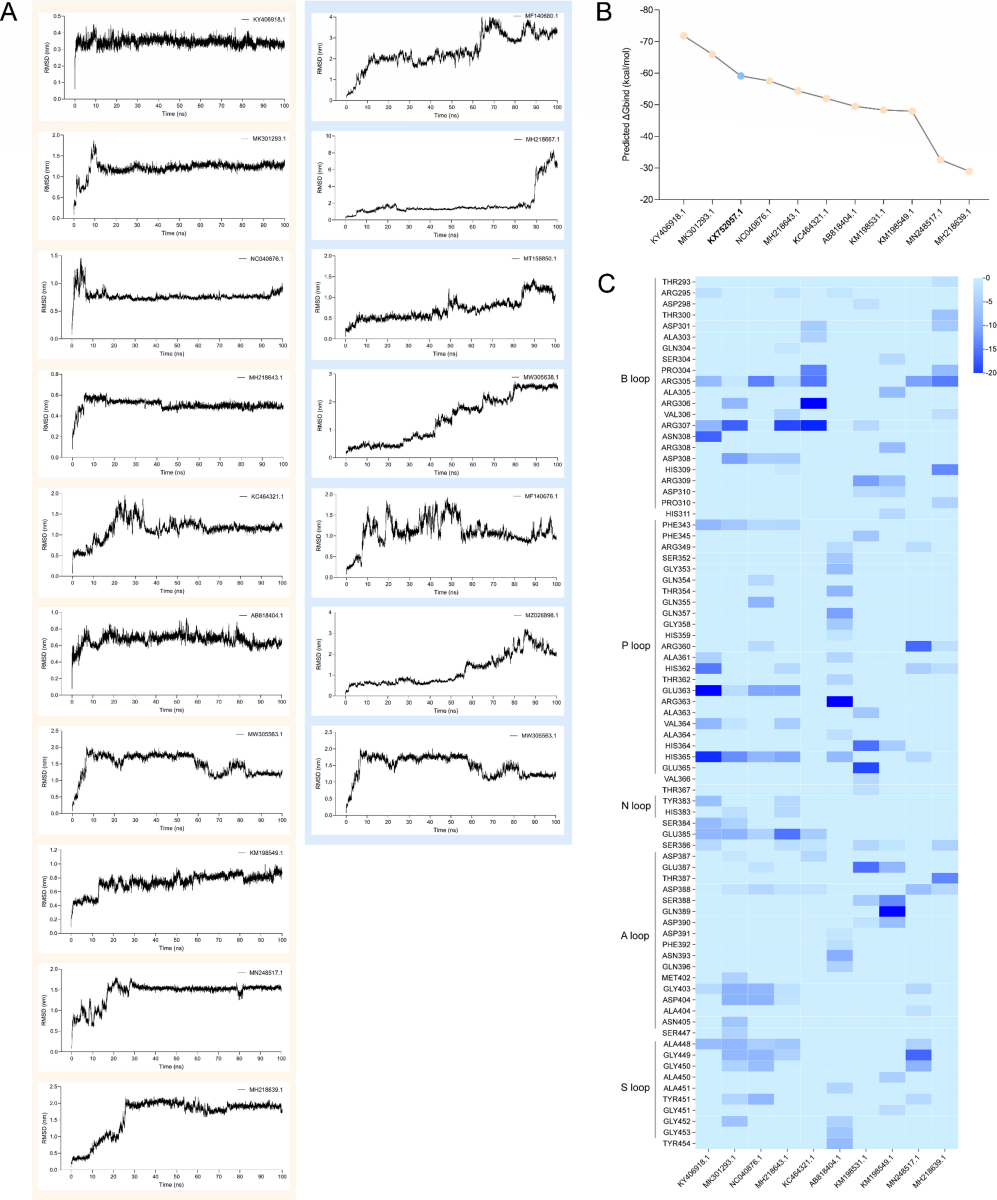
Genotype-dependent differences in Psl binding among representative GII.6 B-loop variants revealed by molecular dynamics and MM-PBSA analyses. (**A**) RMSD trajectories of representative GII.6 norovirus variant P-Psl complexes during 100 ns MD simulations. Variants with yellow backgrounds represent conformationally stable complexes, whereas those with blue backgrounds indicate unstable conformations. (**B**) Predicted binding free energies (ΔG_bind) of 10 representative GII.6 norovirus variants calculated using the MM-PBSA method; the reference variant KX752057.1 is shown in blue. (**C**) Residue-level MM-PBSA energy-decomposition heatmap illustrates the contributions of individual residues to total binding energy.

To investigate the binding differences between various GII.6 strains and the Psl polysaccharide, a total of 86 GII.6 sequences containing full-length VP1 were analyzed, and 17 representative variants were selected for MD simulations based on the polymorphism of amino acids within the B-loop region. These selected variants shared sequence similarities ranging from 83% to 99% with the reference strain KX752057.1 (Table 1). According to the RMSD results of the protein-Psl complexes (Fig. 5A), 10 of the 17 simulation systems reached equilibrium.

**TABLE 1:** Sequence alignment of the B-loop region (residues 290–307) among representative GII.6 norovirus variants^*a*^

GII.6 variants	Identity (%)	B-loop amino acid sequence																				
		290	291	292	293	294	295	296	297	298	299	300	301	/	/	/	302	303	304	305	306	307
KX752057.1	100	I	S	Q	T	A	R	A	V	D	S	T	D	–^*b*^	–	–	S	P	Q	R	A	R
KY406918.1	90	I	S	Q	T	S	R	S	A	D	S	T	D	–	–		S	A	P	R	A	R
MK301293.1	93	I	S	Q	T	S	R	S	S	D	S	T	D	–	–	–	S	A	P	R	R	R
NC040876.1	93	I	G	Q	T	S	R	S	S	D	S	T	D	–	–	–	S	A	P	R	R	R
MH218643.1	91	I	S	Q	T	S	R	S	A	D	S	T	D	–	–	–	S	A	Q	R	V	R
KC464321.1	92	I	G	Q	T	S	R	S	P	D	S	T	D	–	–	–	S	A	P	R	R	R
AB818404.1	99	I	S	Q	T	A	R	A	A	D	S	V	D	–	–	–	S	P	Q	R	A	R
KM198531.1	89	I	G	Q	T	S	R	S	P	D	–	L	D	S	T	D	S	A	P	R	R	R
KM198549.1	89	I	G	Q	T	S	R	S	P	D	–	S	D	S	T	D	S	A	P	R	R	R
MN248517.1	89	V	S	Q	T	S	R	S	A	D	S	T	D	–	–	–	S	A	S	R	V	R
MH218639.1	89	I	S	Q	T	S	R	S	A	D	S	T	D	–	–	–	S	A	P	R	V	R
MF140680.1	83	V	R	Q	E	S	R	S	A	D	S	V	D	S	V	D	S	A	R	R	T	M
MH218667.1	92	I	G	Q	T	S	R	T	S	D	S	T	D	–	–	–	S	A	P	R	R	R
MT158850.1	99	I	S	Q	T	A	R	A	A	D	S	T	D	–	–	–	S	A	P	R	V	R
MF140676.1	84	V	R	Q	E	S	R	S	A	D	S	V	D				S	A	R	R	A	M
MZ026898.1	90	I	G	Q	T	S	K	S	P	D	S	–	D	S	T	D	S	A	P	R	R	R
MW305563.1	90	I	G	Q	T	S	R	S	A	D	S	T	D	–	–	–	S	A	P	R	A	R
MW305638.1	90	I	S	Q	T	S	R	S	A	D	S	T	D	–	–	–	S	V	P	R	A	R

^*a*^ Amino-acid sequences of the B-loop region (residues 290–307) were aligned among 17 representative GII.6 norovirus variants, with the reference variant KX752057.1 included for comparison. Sequence identity (%) represents the overall VP1 nucleotide similarity relative to the reference variant.

^*b*^ The symbol "–" indicates an amino acid deletion at the corresponding sequence position.

The MM/PBSA binding free energy (ΔG_binding) analysis revealed that two strains (KY406918.1 and MK301293.1) exhibited lower binding free energies than the reference strain KX752057.1 (-59 kcal/mol), indicating stronger binding stability with Psl. In contrast, two other strains (MN248517.1 and MH218639.1) showed higher binding free energies than the reference, suggesting weaker interactions with Psl (Fig. 5B).

Further binding free energy decomposition analysis (Fig. 5C) demonstrated that the B-loop region remains the key structural determinant mediating the interaction between GII.6 P proteins and Psl. Interestingly, compared with the reference strain KX752057.1, certain variants also exhibited notable energy contributions from their P-loop and S-loop regions, further highlighting the polymorphic nature of GII.6-Psl interactions.

## DISCUSSION

The distinct binding capacities of GII.6 and GII.4 toward the Psl exopolysaccharide can be largely attributed to their structural and dynamic differences in the P domain. Structural comparison revealed that GII.6 contains an 11 amino acid insertion within the B loop relative to GII.4, along with sequence variations in the B- and T-loop regions (Fig. 1). These differences markedly reshape the P2 subdomain surface, generating a more protruded top region in GII.6. Molecular docking analysis confirmed that this protrusion forms a broader and more favorable binding interface with Psl (Fig. 2). Furthermore, molecular dynamics simulations showed that GII.6 P proteins exhibit greater conformational flexibility than GII.4 (Fig. 3A), which may facilitate dynamic accommodation during ligand recognition. Interface analysis indicated that the GII.4-Psl complex is less stable than GII.6 (Fig. 3C), consistent with MM-PBSA energy decomposition results showing that key residues within the B loop of GII.6 contribute most to the binding free energy (Fig. 3D). The absence of these residues in GII.4 shifts the interaction toward the loop-spacer regions, thereby weakening binding stability. Collectively, these findings suggest that structural insertions and enhanced loop flexibility endow GII.6 with a polymorphic and adaptive binding pattern toward Psl, distinguishing it from the GII.4 strain analyzed in this study. It should also be noted that GII.4 P domains are highly diverse, and strain-dependent binding differences may exist among different GII.4 variants (33). Therefore, the results obtained from the single GII.4 strain used in this study should not be generalized to all GII.4 strains.

Noroviruses exhibit extensive genetic diversity, largely due to their rapid evolutionary rate and frequent intragenotypic recombination events driven by strong selection pressures and high mutation frequencies (34). This genetic diversity is a major driver of viral adaptation and outbreak emergence. Among GII.6 strains, most nucleotide substitutions are synonymous, resulting in minimal alterations to the capsid protein (VP1) sequence. This pattern suggests that the evolution of VP1 in these strains is subject to strong structural or functional constraints (35). Consistent with this, previous studies have demonstrated that three GII.6 variants share similar binding affinities to salivary HBGAs, indicating a conserved recognition mechanism and potential cross-blocking specificity across strains (36). However, our findings suggest that the binding characteristics of Psl differ markedly from those of HBGAs (Fig. 3). Even a single amino acid substitution on the exposed surface of GII.6—such as V297A—can significantly reduce its binding affinity to Psl (Fig. 4). This observation implies that Psl recognition depends on distinct structural determinants compared with HBGA binding. We hypothesize that different GII.6 variants may have evolved distinct adaptive strategies—some preferentially interacting with host-derived HBGAs, while others exhibit affinity toward bacterial polysaccharides such as Psl—both types of ligands potentially contributing to the ecological adaptation and persistence of the GII.6 genotype (Fig. 5). The significance of residue 297 has been predominantly reported in GII.4 norovirus strains (37). A strong correlation has been observed between mutations at residues 297 and 372, suggesting that a substitution at one site is often associated with changes at the other. These two residues exhibit strong signals of co-evolution during the diversification of GII.4 noroviruses (38). Structurally, residues 297 and 372 are in proximity on the viral capsid surface and are located within a key region implicated in determining viral antigenicity. Notably, double mutations at positions 297 and 372 have been shown to abolish binding by mouse-derived monoclonal antibodies (39). Notably, although residue 372 did not appear to contribute significantly to the binding free energy in the interaction heatmap between GII.6 norovirus and Psl (Fig. 3), its close spatial proximity to residue 297 suggests that it may still influence Psl binding indirectly, possibly through conformational modulation. Therefore, future mutational studies targeting residue 372 in GII.6 VP1 may help to further elucidate its potential cooperative role in viral immune evasion and environmental adaptation.

Our sequence logo analysis revealed that approximately 65% of GII.6 strains possess alanine (A) at residue 297 (Fig. 4). Although this residue is highly conserved among circulating GII.6 variants, these strains can still cause sporadic infections and even regional outbreaks (40, 41), indicating that the presence of alanine at position 297 does not completely abolish receptor-binding activity. To further elucidate this phenomenon, we performed molecular dynamics simulations on multiple representative GII.6 P domain-Psl complexes (Fig. 5). The results showed that when residue 297 was alanine, the Psl-binding ability remained detectable, and several strains even exhibited stronger binding stability than the reference strain KX752057.1. MM-PBSA energy decomposition revealed that the differences in binding strength among GII.6 variants were mainly attributed not only to the B loop but also to variations in the P and A loops (Fig. 5). These structural regions have also been identified as critical recognition sites for ligands such as citrate, galactose, and HBGAs (42–44). These findings indicate that GII.6-Psl binding is likely mediated by cooperative interactions among multiple structural domains rather than a single dominant residue. Such multi-site interactions may endow certain GII.6 variants with broader glycan-binding versatility and enhanced environmental persistence.

MD simulations were performed to explore the interaction between the norovirus P domain and Psl. Unlike traditional structural approaches such as X-ray crystallography and cryo-EM, which provide static snapshots, MD simulations yield dynamic trajectories that capture conformational fluctuations under near-physiological conditions. RMSF analysis highlighted the crucial role of loop flexibility in receptor recognition, consistent with previous findings that excessive rigidity can restrict norovirus binding (45). To quantify binding strength, the MM-PBSA method was applied to decompose the total binding free energy into van der Waals, electrostatic, and solvation contributions (46, 47). MM-PBSA analysis indicated that the A294S mutation caused minimal change in binding free energy compared with the wild type (Fig. 4F), whereas experimental results showed a reduced binding affinity to Psl (Fig. 4G). RMSF analysis further revealed that A294S slightly decreased the flexibility of the B loop, thereby impairing conformational adaptation during ligand recognition (Fig. 4C). It should be noted that ELISA provides more direct experimental evidence of binding differences, whereas MM-PBSA reflects the relative binding energetics of a simplified structural model. Therefore, the computational results are better interpreted as supporting the overall trend and providing mechanistic insight, rather than quantitatively matching the magnitude of the ELISA signal. These findings underscore the need to integrate free-energy calculations with dynamic parameters such as RMSD, RMSF, and hydrogen-bond networks to accurately interpret the effects of mutations on protein-ligand interactions.

Polysaccharide structure elucidation generally requires integrated analysis of monosaccharide composition, molecular weight, glycosidic linkage patterns, and branching features, among which the determination of glycosidic linkages is particularly challenging. For Psl from *P. aeruginosa* PAO1, the repeating-unit composition and related structural features have been reported previously (48). In recent years, with the development of artificial intelligence, computational tools such as CandyCrunch have also been developed to assist glycan structure prediction from LC-MS/MS data (49). Nevertheless, this study has certain limitations. The structure of the Psl polysaccharide produced by *P. composti* ODT-54 has not yet been elucidated, and the Psl model used here was constructed based on the reported monosaccharide composition of *P. aeruginosa* PAO1 (Fig. 2). The actual glycosidic linkages and branching architecture in *P. composti* remain unclear. In addition, the simulations employed a Psl monomer rather than the native polysaccharide chain (Fig. 2), which may not fully reflect the multivalent and cooperative interactions occurring under physiological conditions. Future studies should therefore resolve the precise structure of *P. composti*-derived Psl and perform extended simulations involving longer polysaccharide fragments or multiple P domains to better approximate the native binding environment.

In summary, our study demonstrates that the binding affinity between the GII.6 norovirus P protein and Psl is significantly affected by mutations at residue 297, suggesting that bacterial extracellular polysaccharides may play an important role in viral environmental accumulation and transmission. This finding provides new insights into the ecological adaptation and transmission mechanisms of noroviruses.

## SUPPLEMENTAL MATERIAL

Figure S1 aem.02282-25-s0001.docx HPGPC chromatogram of the purified Psl exopolysaccharide.

ASM does not own the copyrights to Supplemental Material that may be linked to, or accessed through, an article. The authors have granted ASM a non-exclusive, world-wide license to publish the Supplemental Material files. Please contact the corresponding author directly for reuse.
