# Supplementary material for: A single amino acid in the viral capsid makes a major contribution to the interaction between human norovirus GII.6 and the bacterial Psl exopolysaccharide
Source: Appl Environ Microbiol. 2026 Jun 22;92(7):e02282-25. doi: 10.1128/aem.02282-25 (PMC13390391; doi:10.1128/aem.02282-25)

**Supplementary materials**

**A single amino acid in the viral capsid makes a major contribution to the interaction between human norovirus GII.6 and the bacterial Psl exopolysaccharide**

Xiang Li^1^, Haoyuan Tian^1^, Yongjie Wang^1,2,3^, Yongxin Yu^1,3^

^1^College of Food Science and Technology, Shanghai Ocean University, Shanghai, China

^2^Laboratory for Marine Biology and Biotechnology, Qingdao Marine Science and Technology Center, Qingdao, China

^3^Laboratory of Quality and Safety Risk Assessment for Aquatic Products on Storage and Preservation (Shanghai), Ministry of Agriculture and Rural Affairs, China

**Figure S1. HPGPC chromatogram of the purified Psl exopolysaccharide.**

The chromatogram showed a predominant peak at 41.45 min. The ratio of weight-average molecular weight (Mw) to number-average molecular weight (Mn) was close to 1, indicating a relatively narrow molecular weight distribution and good overall homogeneity. The molecular weight calibration curves used for calculation were: peak molecular weight (Mp): y = -0.191x + 11.521, R² = 0.9986; Mw: y = -0.1932x + 11.594, R² = 0.9981; Mn: y = -0.1923x + 11.557, R² = 0.998.


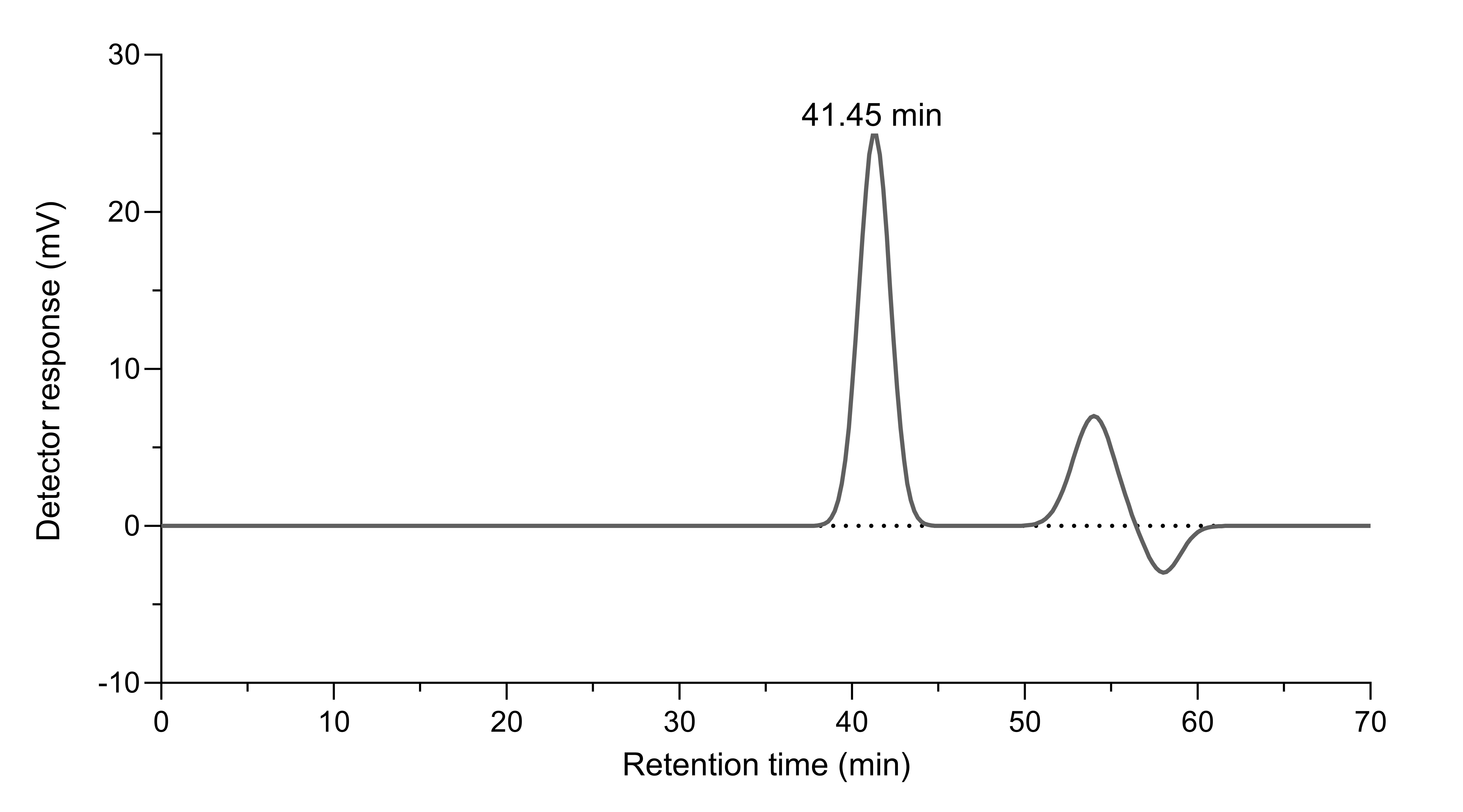

Supplement: Figure S1 — HPGPC chromatogram of the purified Psl exopolysaccharide. [file aem.02282-25-s0001.docx]
